# Trends in retail sales of insecticide-treated nets and untreated nets in Tanzania: cross-section surveys

**DOI:** 10.1186/s12936-023-04726-9

**Published:** 2023-10-04

**Authors:** Benjamin Kamala, Deo Mwingizi, David Dadi, Dana Loll, Peter Gitanya, Charles Mwalimu, Frank Chacky, Stella Kajange, Sara Malima, Mwinyi Khamis, Raya Ibrahim, Naomi Serbantez, Lulu Msangi, Hannah Koenker

**Affiliations:** 1 USAID Tanzania Vector Control Activity, Johns Hopkins University Center for Communication Programs, Dar es Salaam, Tanzania; 2 Muhimbili University of Health and Allied Sciences, Dar es Salaam, Tanzania; 3 USAID Tanzania Vector Control Activity, Johns Hopkins University Center for Communication Programs, Baltimore, MD USA; 4 Tanzania National Malaria Control Program, Ministry of Health, Dodoma, Tanzania; 5 President’s Office- Regional Authority and Local Government, Dodoma, Tanzania; 6 Zanzibar Malaria Elimination Program, Zanzibar, Tanzania; 7 U.S. President’s Malaria Initiative, USAID, Dar es Salaam, Tanzania; 8 USAID Tanzania Vector Control Activity, Tropical Health, Baltimore, MD USA

**Keywords:** Insecticide-treated net, Counterfeit, Retail survey, Leaked nets, Commercial sector

## Abstract

**Background:**

The commercial sector plays a vital role in mosquito net ownership and access in Tanzania. The National Malaria Strategic Plan (NMSP) includes long-lasting insecticidal nets (LLIN) delivery through the commercial sector as a complementary mechanism. The NMSP aims to increase LLIN sales while decreasing untreated mosquito net sales. This survey aimed to track quantities, market share of different net categories, prices, and origins of mosquito nets in retail markets and to engage stakeholders to analyse market trends.

**Methods:**

This mixed-method mosquito net retail outlet survey was conducted in mid-2021 in six and in mid-2022 in eight regions. Field teams identified net-selling outlets in major urban and peri-urban markets and used snowball sampling to identify additional outlets. A structured questionnaire was used, and photos of available mosquito net products were taken. Key informant interviews were conducted with wholesalers and retailers. The relative market share of a product was calculated by using the mean of each sales category as frequency weights. Qualitative data analysis was undertaken by summarizing common themes and observations based on the research question.

**Results:**

A total of 394 and 1139 outlets were surveyed in 2021 and 2022, respectively. More than 96% of distributed brands in both years were untreated nets. The market share for untreated mosquito nets was 99.2% in 2021 and 88.3% in 2022. Bed net sales were seasonal, peaking in the rainy season and at the start of the school year. Leaked LLINs from the public sector comprised 0.3% of the market share in 2021 and 8.3% in 2022. Kigoma markets had the most significant frequency of leaked LLIN products. Legitimate LLINs were rare in 2021 (n = 2) and not found in 2022, despite the presence of a local LLIN manufacturer. A small number (n = 3) of untreated nets fabricated in China claiming to be LLINs were observed in 2022.

**Conclusions:**

Despite NMCP’s strategic approach to increasing retail market share for legitimate LLINs, significant challenges remain. Efforts are needed to change the current situation given the context of large-scale public sector distributions of LLINs, the higher consumer cost of LLINs, the lack of bed net varieties. Improvement of registration process is recommended.

**Supplementary Information:**

The online version contains supplementary material available at 10.1186/s12936-023-04726-9.

## Background

The commercial sector in Tanzania has played an important role in bed net ownership and access even before the launch of subsidized bundled insecticide treated-bed nets (ITNs) in 2004 through SMARTNET and Tanzania National Voucher Scheme (TNVS) projects which provided treated nets to pregnant women and infants [1–3]. Since the introduction of long-lasting insecticidal nets (LLINs) mass campaigns in 2007, however, public sector free distributions provide the majority of bed nets to households. Within the Tanzanian commercial bed net market, untreated nets dominate, accounting for about 75% of the market share in 2017 [4]. Untreated bed nets comprised 9.3% of all nets observed during the 2017 Tanzania Malaria Indicator Survey (TMIS), ranging from 2% in Kigoma to 12% in Katavi [5]. While recognizing that untreated bed nets can fill some gaps in household net access without exacerbating insecticide resistance, the goal of the National Malaria Control Programme (NMCP) is to increase long-lasting insecticidal net (LLIN) sales while decreasing untreated net sales. The TMIS also reported that nationwide, 16.6% of bed nets (both LLIN and untreated) were reported by survey respondents as purchased, ranging from less than 5% in Songwe and Njombe to over 40% in Dar es Salaam. In Zanzibar, 21% of bed nets in the Mjini Magharibi region had been purchased, while less than 5% in the other four mainland regions had been purchased.

The Tanzania National LLIN Strategy for Mainland includes a commercial sector component, reflecting the high ownership rates of purchased nets, particularly in urban areas. Most of these purchased nets are untreated or, if treated, are likely to be “leaked” nets from public sector distributions in and/or surrounding countries [4]. On the mainland, one of the NMCP’s stated objectives is to increase LLIN sales to 1.5 million per year while decreasing untreated net sales to 300,000 per year, reversing the current ratio to complement free public sector distributions. Subsequently, the NMCP aims to build the sales of LLINs to between 2 and 3 million per year by accelerating product registration, supporting healthy competition, building demand for LLINs over untreated nets, optimizing distribution chains, and minimizing or preventing detrimental influences such as public sector leakage or counterfeit nets. In contrast to mainland Tanzania, the Zanzibar Malaria Elimination Programme (ZAMEP) LLIN strategy is to provide free LLINs to all community members; private markets are allowed to sell nets, but there is no formal commercial LLIN strategy.

Landscaping of LLIN national registration processes conducted in 2019 [6, 7] found that Tanzania was one of the five countries that require LLIN manufacturers to conduct local, full, or semi-field trials to confirm the effectiveness of LLIN products. Tanzania also had one of the longest timelines for product registration (7–12 months), second only to South Africa. As of 2022, only seven of the 25 LLIN products prequalified by the World Health Organization (WHO) were listed as having completed LLIN registration with the Tropical Pesticide Research Institute (TPRI) [8], which oversees registration for all agricultural and public health pesticides for the country. TPRI was renamed in 2022 and is now the Tanzania Plant Health and Pesticides Authority (TPHAPA). Tax and tariff policies for mosquito netting have historically favoured the importation of untreated net compared to treated netting, making local “cut and sew” production of LLINs less cost-effective. Tanzania, nonetheless, has the continent’s only factory producing LLINs for local and international markets under a licensing agreement with Sumitomo Chemical.

There is a need to track quantities, prices, and origins of nets, engage stakeholders to analyse market trends and use the data to inform interventions to shape the market to favor increased sales of LLINs. The NMCP and ZAMEP thus embarked on a market research study in collaboration with selected market associations in Tanzania, regulatory bodies, and the USAID-funded Tanzania Vector Control Activity (TVCA). The primary objectives of the market survey were to annually assess the magnitude of bed net sales at key retail outlets across Tanzania and assess market share among treated and untreated bed nets. Secondary objectives included identifying types of existing counterfeiting, misleading, or leaked LLIN violations in Tanzania, and clarifying whether the source of the breach was domestic or international, where possible.

## Methods

### Study sites

**Fig. 1:**
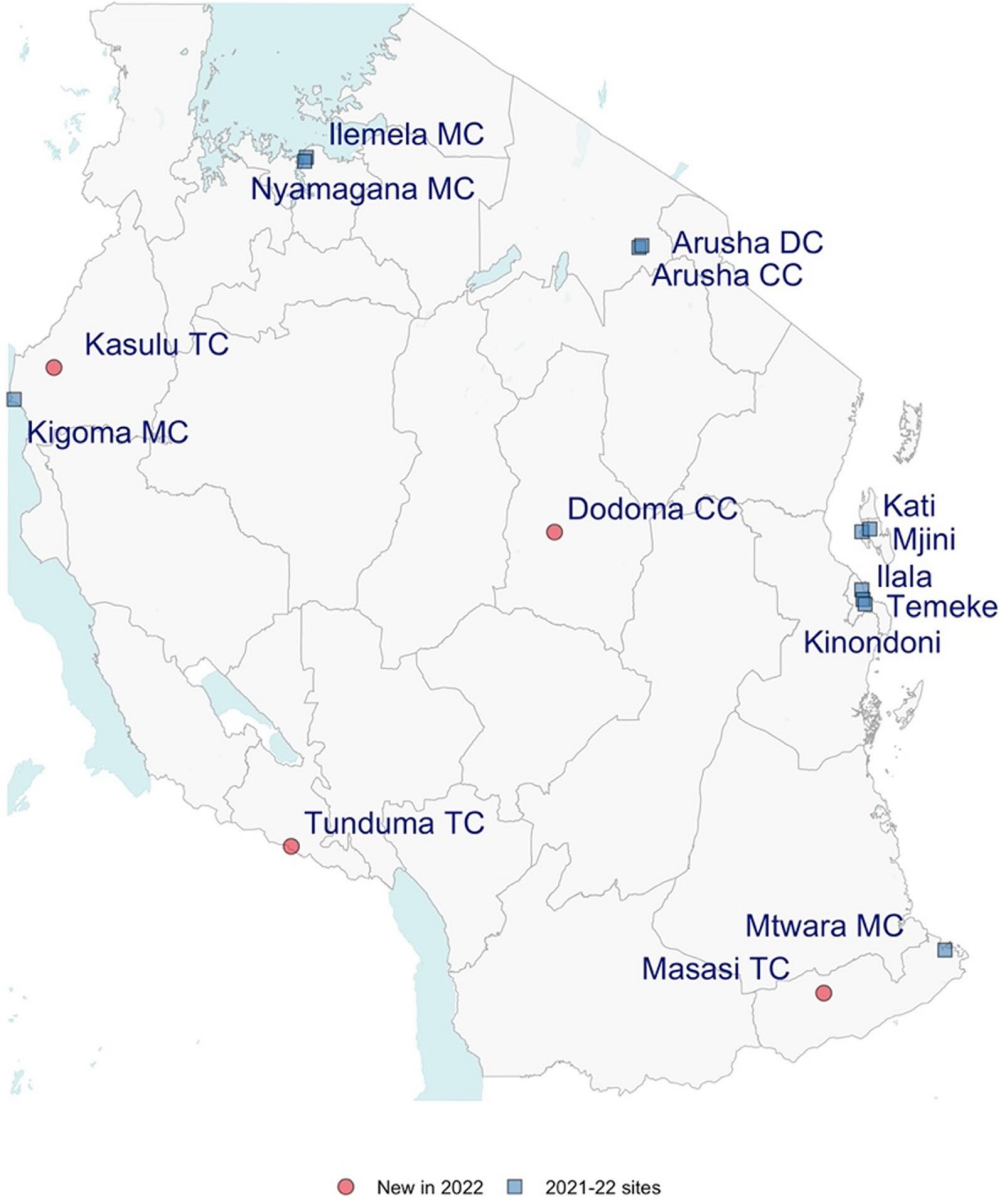
Map of retail bed net market sites, 2021 and 2022

Major urban areas within mainland Tanzania and Zanzibar were selected purposely, with six sites in 2021 and eight in 2022. In 2021, each site comprised only one council, while in 2022, the additional two sites each comprised two councils. The selection criterion was to include the major markets for nets and LLINs. The selected locations are shown in Fig. 1.

### Study design, sampling, data collection, and analysis

The basic design was an exploratory, observational study using quantitative and qualitative data collection methods. Within each site, up to three local markets were purposely selected based on consultation with the local Ministry of Health, local government, and regional authority staff. At least one identified market in each site was classified as urban or peri urban. Within each of the councils, several sub-markets were identified; the majority of these were urban markets. Additional File 1: Table S1 details regions, councils (mainland) or districts (Zanzibar), and the number of markets per council/district.

Within each market, the team purposely selected several outlets with the intention of including a diversity of net-selling outlets. An outlet was defined as an established area with bed net retail sales. Field teams randomly started at first identified net selling outlets and then moved through the market until the target for each outlet type was reached. An adaptive approach was used in some cases to capture the specific outlets, such as mobile vendors who often only sell later in the day during rush hours or along main streets or junctions.

Research assistants were trained in qualitative interviewing techniques, survey administration, research ethics, and recognition of various LLIN and untreated net brands as well as suspicious and counterfeit LLIN products. A visual aid to help identify suspicious nets was used. Local authorities and relevant market associations were informed of the study and its objectives before fieldwork. Fieldwork was carried out between April and June in both 2021 and 2022. Research assistants collected information about net products, sales volumes, and photos using a structured questionnaire on Android tablets with Kobo Toolbox, an Open Data Kit (ODK)-based software for mobile data collection. This included taking pictures of the back and front of each net product and capturing details of manufacturer or insecticide content. One sample of each LLIN product was procured at market price for later detailed inspection. Data were sent to a secure server daily and screened, with immediate feedback to the field teams as necessary. Data were then imported into Stata 17 for quality and consistency checks. Finally, each net product was screened based on the pictures taken in addition to the information collected. Characteristics of specific products were compared within sites, and a final classification was made according to the following five category definitions based on WHO recommendations for medicines, the first three of which represent the “problematic” net products targeted by the study:

- Counterfeit nets: products that fraudulently imitate an existing LLIN brand either as a direct copy or by presenting similarly to the original but with a slightly different name (knock-off).
- Misleading nets: an untreated net in packaging that suggests or implies the net is insecticidal or treated with insecticide.
- Leaked LLIN: an LLIN with signs that the public sector originally procured it for free distribution.
- Legitimate LLIN: WHO pre-qualified LLIN brands without any indication that they are not from the original manufacturer and with no signs of procurement through the public sector.
- Untreated nets: nets that do not claim to be insecticide-treated.

During data evaluation, the batch numbers and packaging of LLIN products were used to confirm the originally intended destination, year of manufacture, and relevant stakeholders (manufacturers or net distribution implementers) to allow definite categorization of net products. To assess a product’s relative market or sales share, the analysis was weighted by the reported sales in the last 3 months by using the mean of each sales category as frequency weights.

Key informant interviews (KII) were conducted with wholesalers and retailers within a given site. Retailers within each site identified wholesalers. Interviewers were held in a private room, and hand-written notes were taken. Notes were then summarized in English and typed up for analysis. Qualitative analysis was undertaken by an experienced analyst by screening all notes and summarizing common themes and observations based on the research question within and across sites.

## Results

In 2021, teams visited a total of 58 markets with 394 outlets and observed 745 products. In 2022, teams visited a total of 108 markets with 1139 outlets, observing 1698 products. Outlets included pharmacies, market stalls, convenience shops, supermarkets, and mobile vendors. Very few pharmacies and supermarkets carried bed nets, comprising less than 5% of all outlets sampled. In each site, the total number of outlets interviewed ranged from 1 (Kati in Zanzibar) to 103 (Ilala in Dar es Salaam).

**Fig. 2:**
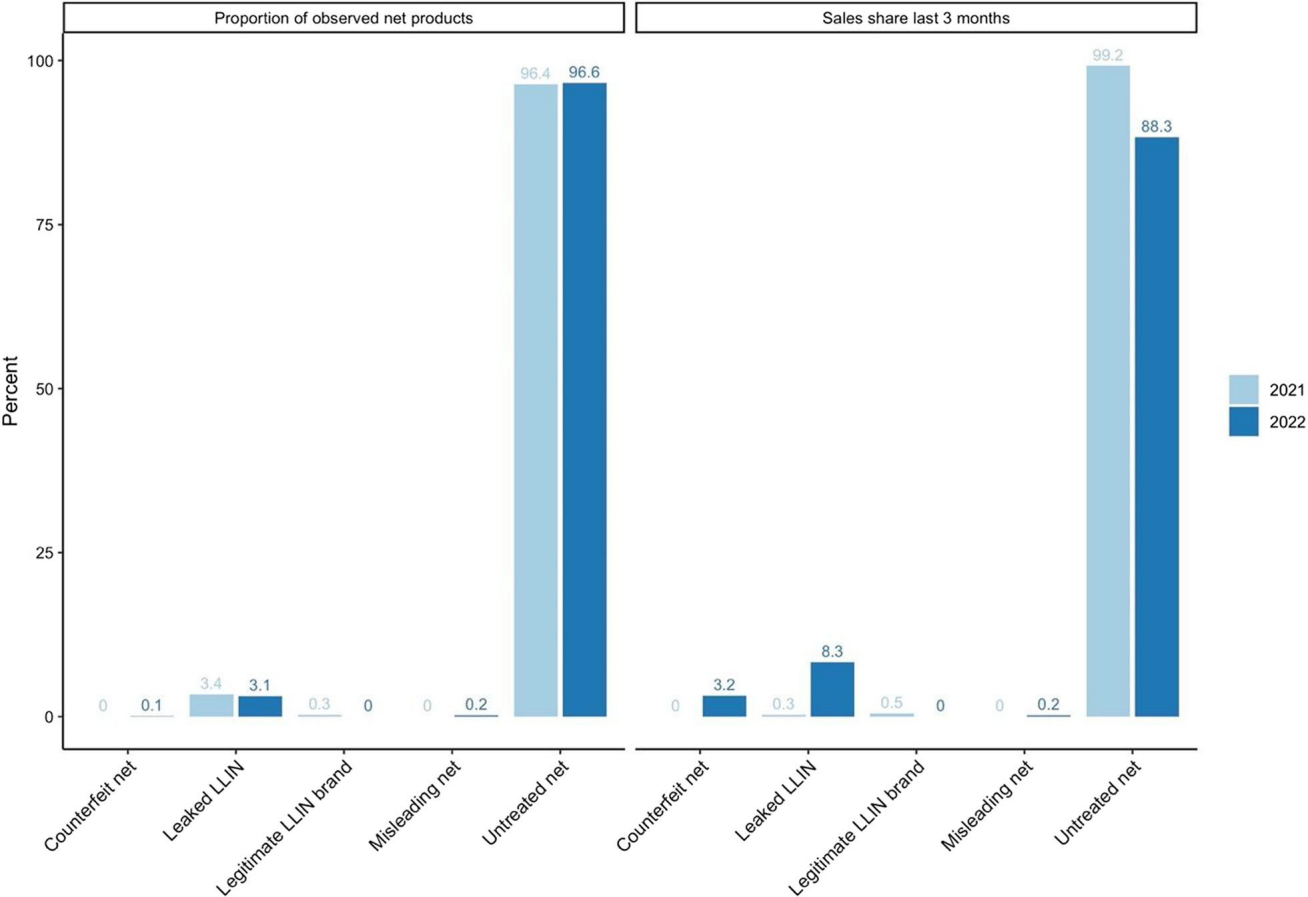
Distribution of net types among all products (left) and sales share in the past 3 months (right)

In both 2021 and 2022, 97% of observed net products were untreated (Fig. 1). No counterfeit or misleading nets were found in the surveyed outlets in 2021. In 2022, two counterfeit and three misleading nets were found in the surveyed outlets. The market share for untreated mosquito nets was 99.2% in 2021 and 88.3% in 2022. Kigoma had the greatest frequency of leaked LLIN products, although most net types observed were still untreated for both 2021 and 2022. In 2022 leaked nets (mostly in Kigoma) comprised 8.3% of the market share. The type of net and their share of reported sales in the last 3 months is presented in Fig. 2.

### Distribution of brand among sales share in the past 3 months: 2021 (n = 74,005) & 2022 (n = 111,005)

**Fig. 3:**
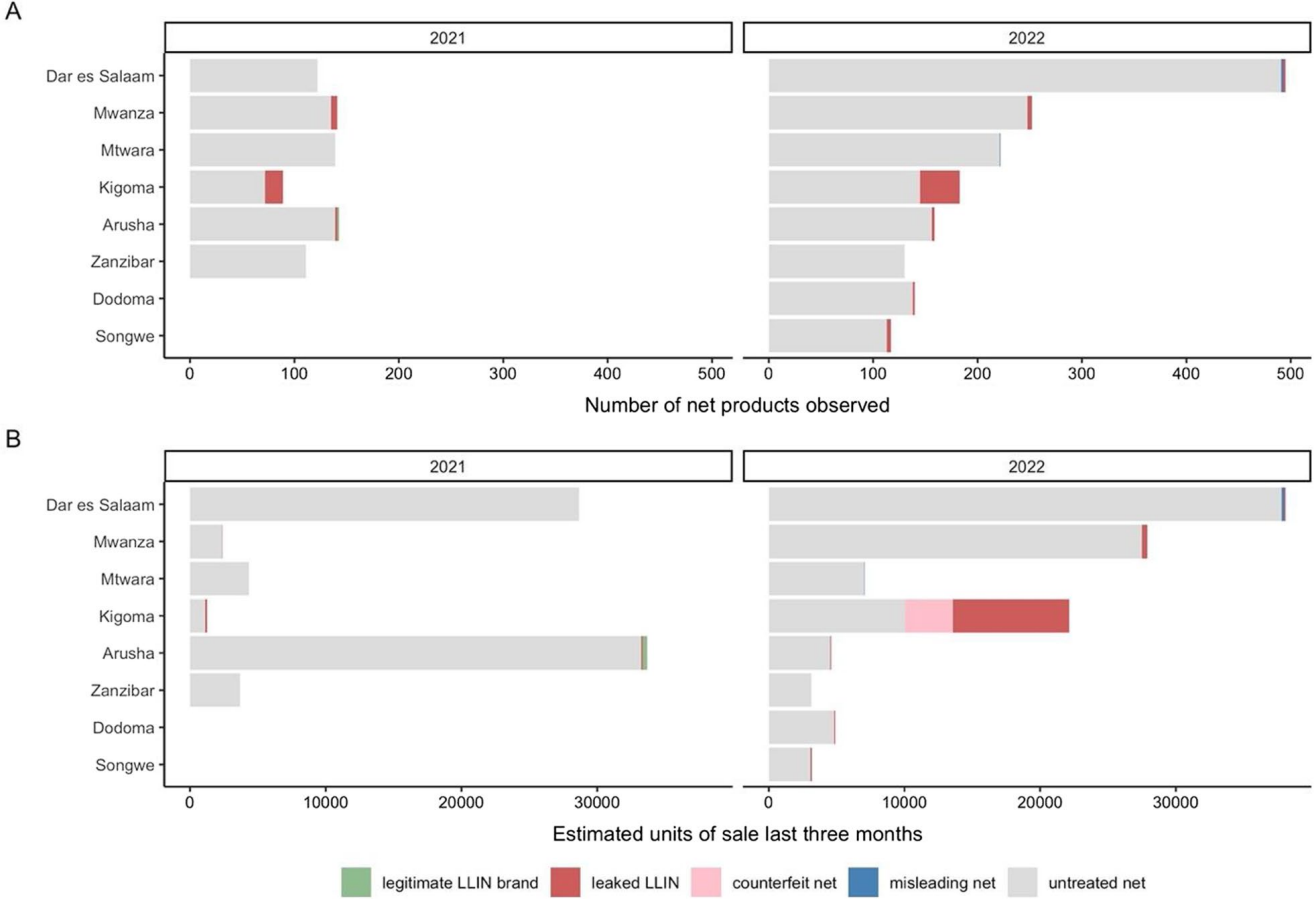
Numbers of net products observed (**A**) and estimated units of sale in the past 3 months by type of net for 2021 and 2022

The vast majority of nets were untreated in the two surveys, both for observed products and for sales share in the previous 3 months. Figure 3 shows these findings disaggregated by study site, year, and net type. In 2021, Kigoma had a higher proportion of leaked LLINs; other regions have nearly zero values. In 2022, in addition to Kigoma, Arusha, and Mwanza had some leaked LLINs. In terms of market share, in 2021, almost all market shares were occupied by untreated nets. But in 2022, the leaked and counterfeit net brands reportedly had high sales volumes; thus, when market share is considered, just over half of all Kigoma sales in the last 3 months were for leaked (39%) or counterfeit (16%) LLINs. Notably, a Yorkool LLIN repackaged as a PermaNet 2.0 had a reported sales volume of 1001–5000 units, driving the results shown in Fig. 2. In all regions, untreated nets dominated the market, with Kigoma recording most sales of leaked LLINs, including PermaNet, Yorkool (repackaged as PermaNet 2.0), OlysetPlus, DuraNet (via Kenya), and Yahe (via DRC or Burundi).

Asian-fabricated, untreated nets (nearly all Chinese in origin) dominated the markets in all sites and comprised nearly 90% of overall sales. Tanzanian-made untreated nets, including SafiNet, comprised around 14% of sales in Arusha and less than 10% in other regions.

### Prices

In 2021, regular-size legitimate LLINs had a median price of 10,000 Tanzanian shillings (TZS) (n = 2), leaked LLINs a median price of TZS 6000 (range TZS 3000 to 10,000, n = 24), and untreated nets had a comparable median price of 9000 TZS, (range 4000 to 100,000; IQR 7000–10,000, n = 896) with a few (n = 25) expensive outliers, generally larger decorative Chinese brands, and domed nets. The median price for untreated queen-size nets was 10,000 TZS (range 6000–35,000; IQR 10,000–13,000, n = 57) in 2021; the USD-TZS exchange rate in both 2021 and 2022 averaged 2300 TZS to 1 USD.

**Fig. 4:**
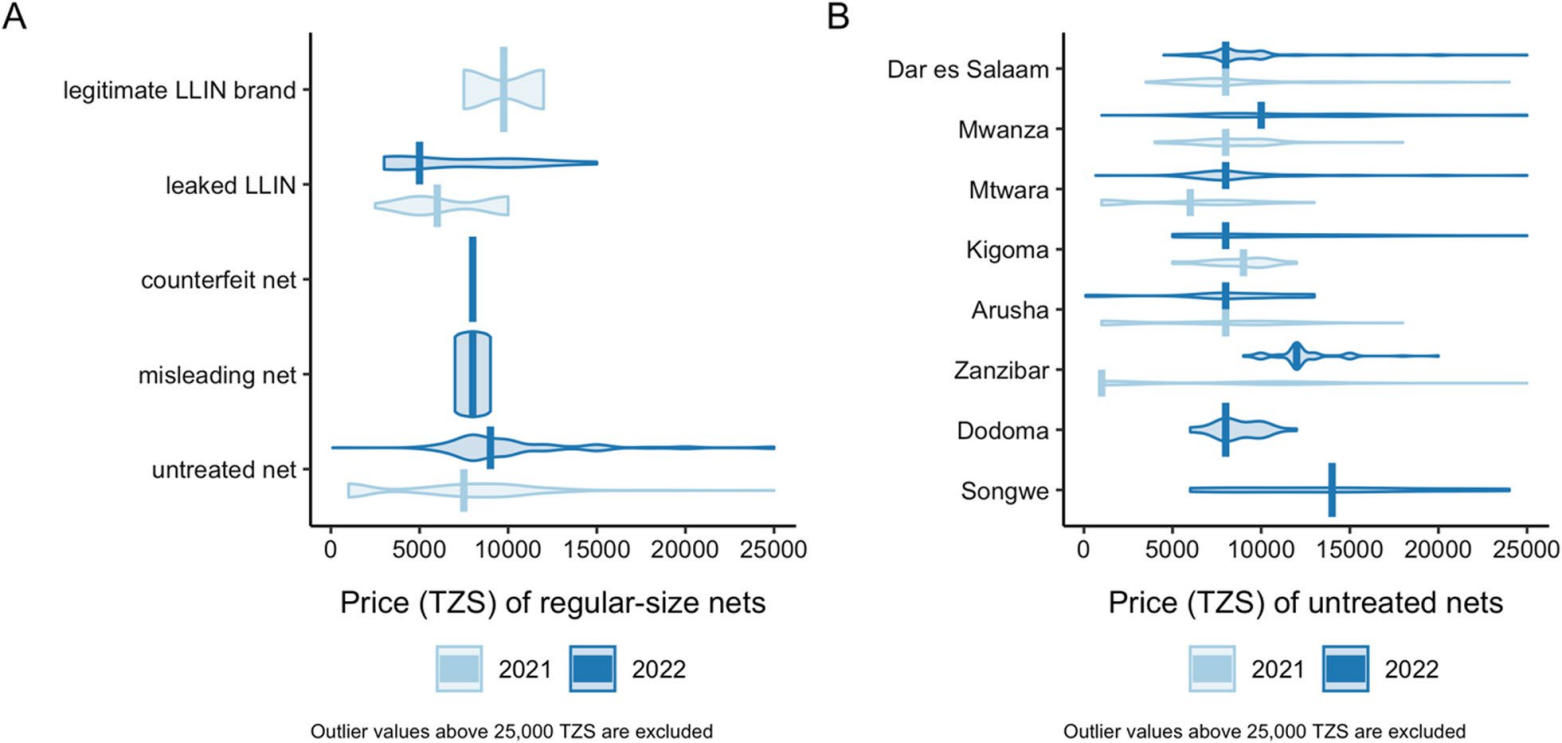
**A** Sales prices of rectangular regular-size nets by net category in Tanzania in 2021 and 2022 surveys; **B** Sales prices of untreated rectangular regular-size nets, by site, in 2021 and 2022 surveys. 1 USD = 2300 TZS.

In 2022, leaked LLINs had a median price of TZS 5000 (range 3000–15,000; IQR 3500–10,000; n = 16) for regular size. The sole counterfeit net product was priced at TZS 8000 for regular size and TZS 10,000 for queen size, while the misleading nets had a median price of TZS 8000 for regular size (range 7000–9000; n = 3) and 9000 for queen (n = 1). Untreated nets had a comparable median price of 9000 TZS for regular size (range 1000 − 120,000; IQR 8000–12,000, n = 896) and 10,000 for queen size (range 800 − 100,000; IQR 8000–15,000, n = 632). Figure 4 A shows median prices for regular (double) size nets from 2021 to 2022 surveys. Median prices for untreated nets across the study sites for 2021 and 2022 are shown in Fig. 4B. The median price for regular-size untreated nets increased since 2021 in Zanzibar, Mwanza, and Mtwara but remained relatively stable in Dar es Salaam, Arusha, and Kigoma. Songwe had the highest median prices for regular-size untreated nets compared to other regions.

#### Qualitative results

In 2021 a total of 34 key informant interviews (KII) were completed across the six sites, including 25 wholesalers and nine retailers. In 2022, a total of 69 KII were completed across the eight regions. In both years, retail outlets offered a range of net shapes (rectangular, conical, and domed) and sizes classified as ‘school’ (individual), ‘regular’ (double), queen, and king-sized. The best-selling nets were brands produced in China, including Afya Net, BO XIN Net, and HM textile nets. Most respondents mentioned these nets were popular due to their affordability, perceived durability, availability, and multiple colors, sizes, mesh size, and shape options. Most wholesalers and retailers lamented that free nets distributed by the government reduced their market share; some retailers saw the government as a market competitor.

The COVID-19 pandemic delayed importations of nets from China due to lockdowns beginning in 2020. Sellers reported that since then, the business has never been the same. Most sellers mentioned that most customers do not ask whether a net is treated; instead, their buying decision is influenced by the texture of the bed net material, bed net size, and mesh size. However, those who differentiate reportedly perceive treated nets to be dangerous for their health and/or to cause irritation to their skin. Some tailors buy plain untreated bed nets from retailers, decorate them, and then re-sell them to the final consumers, retailers, and hawkers at a premium price.

Sellers reported in both 2021 and 2022 that bed net sales show seasonal trends, peaking at the start of the school year for use by students, particularly at boarding schools, and during the rainy season, depending on the region. For example, the Kigoma and Mwanza region informants mentioned that bed nets are sold during rainy seasons and school openings. Conversely, the sellers in Arusha and Songwe noted that most people say that these regions have no mosquitoes because of their colder climatic conditions. Therefore, bed nets are sold primarily to boarding school students. In Songwe, nets were reported to be sold to Zambians.

The majority of the respondents who were found with leaked LLINs did not divulge their suppliers, and those who did cite Kariakoo in Dar es Salaam as their primary source of the suspicious/leaked LLINs but did not specify the exact suppliers. Informants in Kigoma mentioned that some treated bed nets are leaking from some of the supply chain of the public distribution channels in Kigoma region as well as refugee camps but did not specify suppliers.

## Discussion

This study was designed to assess the overall market share for untreated nets and LLINs and to identify any leaked, misleading, or counterfeit LLIN products in markets in eight regions of Tanzania. Overall, untreated bed nets dominate the Tanzanian market. The main reasons for the selection of untreated bed nets were affordability, perceived durability, availability, and multiple options for color, size, mesh size, and shape. Counterfeit and misleading nets were rare; leaked LLINs from free public sector distribution were present but extremely limited. Legitimate LLINs were virtually non-existent in the retail market.

Untreated nets dominated across all markets, comprising 96% of all products observed and 88% of estimated net sales in the 3 months prior to the survey, which is consistent with previous surveys. These nets are cheaper, predominantly polyester, and come in a wide range of sizes, shapes, colors, and decorative options, and their wide availability across major urban markets makes them a top choice for families seeking to add or replace nets in their households. Among the Chinese-manufactured nets, a good number of “dome” nets do not need hanging and appear to be of some consumer interest.

Secondly, the finding that few counterfeit or misleading nets were found in Tanzania in the 2021 and 2022 surveys is consistent with the results from 2017 [4] and is encouraging. Only one counterfeit PermaNet 2.0 product was observed in 2022, along with a Yorkool LLIN repackaged with photocopied PermaNet 2.0 branding. The latter had a reportedly large sales volume. While counterfeit nets have been reported in other settings, notably Nigeria, their presence is still limited. Additional work may be needed to monitor developments in this area.

Thirdly, leaked LLINs from free public sector distribution were present but quite limited at 8% market share in 2022, an increase from 2021 (0.3%) and slightly below 2017 levels when they comprised 11% of the market share in the Dar es Salaam and Mwanza markets. Leaked LLINs originated within Tanzania (Olyset; OlysetPlus) and from neighboring countries (Yahe; Interceptor G2, DuraNet, PermaNet 3.0). Leaked LLINs were found in all markets except Zanzibar and Mtwara. The limited number of leaked nets may imply that current national campaigns and distributions are likely to be well implemented and appreciated as people are not selling their nets to distributors, nor are large numbers of nets being stolen.

Legitimate LLINs were utterly absent in 2022 and have been effectively crowded out from the studied markets. This starkly contrasts Tanzania’s early history of socially marketed LLINs when LLINs for pregnant women and infants were available all over the country from 2005 to 2014 [1–3]. Tanzania began mass distributions of LLINs in 2007 through campaigns [9] and through schools starting in 2013. Still, donor support for the Tanzania Voucher Scheme was withdrawn in 2014, and free LLINs became the norm at antenatal clinics and immunization visits. At the same time, the process for registering LLIN products in Tanzania has for some time been time and resource-consuming relative to other countries, requiring local field evaluations of products that are already WHOPES-recommended or WHO-pre-qualified [6, 7]. Moreover, untreated nets have lower taxes and tariffs compared to treated nets, making investments in retail LLINs less attractive to businesspeople. As of 2022, only seven of the 25 WHO pre-qualified LLINs had completed product registration in Tanzania. With the costs and timelines for product registration relatively burdensome and the market so limited, manufacturers and wholesalers have little to no incentive to invest in retail activities for LLINs. This includes Tanzania’s local manufacturer, whose LLIN products were rare in 2021 and completely absent in 2022.

A 2016 study [10] explored willingness to pay for LLINs over untreated nets in a non-hypothetical choice experiment and found that study participants in Ruvuma and Mwanza regions were willing to pay an additional 2742 TZS (lower two wealth quintiles) for an LLIN compared to a comparable untreated net; participants in the top three wealth quintiles were willing to pay an additional 1704 TZS, indicating that there is demand for LLINs. However, size was more critical than insecticidal treatment; participants in the lower two wealth quintiles were willing to pay 3000 TZS for a larger net vs. a small net. Overall, households with insufficient nets were the most likely to buy a net. However, willingness to pay cannot be acted upon if LLINs are unavailable at local markets.

Given the documented importance of sleeping under LLINs for malaria control, NMCP and ZAMEP have prioritized efforts to increase market share for legitimate LLINs and to reduce market share for untreated nets, including the development of an ITN Commercial Sector Task Force and an associated ITN Commercial Sector Implementation Guideline. However, increasing sales of retail LLINs remains a significant challenge in a context where large-scale public sector distributions of LLINs through mass campaigns, school distribution, and reproductive and child health (RCH) clinics aim to provide universal coverage and product registration processes have deterred retail activities for LLINs. The large market share for untreated nets may reflect, as observed in some KIIs, a desire for larger or more decorative nets, nets that don’t provoke itchiness, or that have a conical shape that is easier to hang. It is also likely that untreated nets play a key role in filling gaps in bed net access at the household level, particularly for households that may not have a pregnant woman, infant, or primary school student or in councils that were not targeted for the 2020 mass replacement campaign. Data from the 2021 Malaria Behavioral Survey and 2021 DHS/MIS will provide more detailed information on ownership of both LLINs and untreated nets within households and will help to inform the overall picture of coverage with both types of nets across Tanzania.

### Limitations

The study had some limitations. First, the sample of markets and outlets was not representative of the whole of Tanzania; this was mitigated by ensuring that the selected markets were the primary hubs of net and LLIN turnover, so it is plausible to assume that the study still captured the general situation correctly. Second, some respondents refused to participate in the survey and KIIs, but these instances were relatively limited. Third, there may have been some misclassification of products into the five categories, particularly for nets with unlabeled packaging. However, these instances were few (n = 24 in 2021 and n = 105 in 2022; 3% and 6% of the total samples, respectively) and are unlikely to affect the overall results.

## Conclusions

Overall untreated bed nets dominate the private market. Leaked public-sector nets were scarce across all markets, and most had originated from distributions in neighboring countries. Tanzania is unlikely to see increased retail sales of LLINs without significant changes in LLIN distribution policy and national regulatory processes for LLIN; it appears that manufacturers are not finding it worthwhile to promote and sell retail LLINs given the ongoing competition of mass distributions of LLINs and lower-cost untreated nets. Manufacturers should be encouraged to have additional options for LLINs so that people can get varied sizes, colors, mesh size, and shape with a net with insecticide. However, untreated nets still provide barrier protection against malaria vectors and contribute to filling household-level gaps in bed net coverage.

### Supplementary Information

**Additional file 1: Table S1.** Regions, councils/districts, and the number of markets per council.

## Data Availability

The datasets used and/or analyzed during the current study are available from the corresponding author on reasonable request.

## Abbreviations

IRB: Institutional Review Board
ITN: Insecticide-treated bed net
KII: Key Informant Interview
LLIN: Long Lasting Insecticidal Net
NIMR: National Institute for Medical Research
NMCP: National Malaria Control Programme
ODK: Open Data Kit
RCH: Reproductive and Child Health
T MIS: Tanzania Malaria Indicator Survey
TNVS: Tanzania National Voucher Scheme
TPHPA: Tanzania Plant Health and Pesticide Authority
TPRI: Tanzania Pesticide Research Institute
TVCA: Tanzania Vector Control Activity
WHO: World Health Organization
WHOPES: WHO Pesticide Evaluation Scheme
ZAMEP: Zanzibar Malaria Elimination Programme

## References

[1] Mushi AK, Schellenberg JR, Mponda H, Lengeler C (2003). Targeted subsidy for malaria control with treated nets using a discount voucher system in Tanzania. Health Policy Plan.

[2] Magesa SM, Lengeler C, de Savigny D, Miller JE, Njau RJ, Kramer K (2005). Creating an enabling environment for taking insecticide treated nets to national scale: the Tanzanian experience. Malar J.

[3] Marchant T, Schellenberg D, Nathan R, Armstrong-Schellenberg J, Mponda H, Jones C (2010). Assessment of a national voucher scheme to deliver insecticide-treated mosquito nets to pregnant women. CMAJ.

[4] Kilian A, Obi E, Koenker H, Dimoso K, Opoku R, Babalola S et al. Presence and frequency of counterfeit and questionable mosquito net products in markets in three African countries – an exploratory study. VectorWorks Project, ohns Hopkins University Center for Communication Programs, Baltimore, MD; 2018.

[5] Ministry of Health, Community Development, Gender, Elderly and Children (MoHCDGEC) [Tanzania Mainland], Ministry of Health (MoH) [Zanzibar], National Bureau of Statistics (NBS), Office of the Chief Government Statistician (OCGS), and ICF. 2017. Tanzania Malaria Indicator Survey 2017. Dar es Salaam, MoHCDGEC, MoH, NBS, OCGS, and ICF. Accessed 17 May 2023

[6] Innovation to Impact. Pan-African registration landscape for vector control tools. https://innovationtoimpact.org/wp-content/uploads/2019/09/Pan-African-Registration-Landscape-for-Vector-Control-Tools.pdf. Accessed 17 May 2023.

[7] Innovation to Impact (2019) Selected country registration processes for vector control tools. https://innovationtoimpact.org/wp-content/uploads/2019/09/Selected-Country-Registration-Processes-for-Vector-Control-Tools.pdf. Accessed 18 May 2023.

[8] Tropical Pesticides Research Institute. List of Registered Pesticides to be used in the Republic of Tanzania. https://www.tpri.go.tz/storage/app/media/uploaded-files/List%20of%20Registered%20Pesticides%20to%20be%20used%20in%20the%20United%20Republic%20of%20Tanzania.pdf. Accessed 19 May 2023.

[9] Bonner K, Mwita A, McElroy PD, Omari S, Mzava A, Lengeler C (2011). Design, implementation and evaluation of a national campaign to distribute nine million free LLINs to children under five years of age in Tanzania. Malar J.

[10] Gingrich CD, Ricotta E, Kahwa A, Kahabuka C, Koenker H (2017). Demand and willingness-to-pay for bed nets in Tanzania: results from a choice experiment. Malar J.

