# Supplementary material for: Trends in retail sales of insecticide-treated nets and untreated nets in Tanzania: cross-section surveys
Source: Malar J. 2023 Oct 4;22:296. doi: 10.1186/s12936-023-04726-9 (PMC10552209; doi:10.1186/s12936-023-04726-9)
Supplement: Supplementary file 1 — Additional file 1: Table S1. Regions, councils/districts, and the number of markets per council. [file 12936_2023_4726_MOESM1_ESM.docx]

# Additional Information

Table S1: Regions, councils/districts, and the number of markets per council.

| 2021 | Region | council/district | Number of Markets Visited |  |
| --- | --- | --- | --- | --- |
| 1 | Dar es Salaam | Kinondoni municipal | 2 |  |
| 2 | Dar es Salaam | Ilala municipal | 5 |  |
| 3 | Dar es Salaam | Temeke municipal | 5 |  |
| 4 | Mwanza | Ilemela municipal | 5 |  |
|  | Mwanza | Nyamagana municipal | 5 |  |
| 5 | Kigoma | Kigoma Municipal | 5 |  |
| 6 | Zanzibar | West B (Magharibi B) | 4 |  |
| 7 | Zanzibar | Zanzibar Town (Mjini) | 7 |  |
| 8 | Arusha | Arusha City | 10 |  |
|  | Mtwara | Mtwara Municipal | 10 |  |
| 9 | Total number of markets visited |  | 58 |  |
|  |  |  |  |  |
| 2022 | Regions | Councils |  |  |
| 1 | Dar-es-salaam | Kinondoni | 6 |  |
| 2 | Dar-es-salaam | Ilala | 6 |  |
| 3 | Dar-es-salaam | Temeke | 5 |  |
| 4 | Unguja | Mjini, Magharibi A&B | 21 |  |
|  |  |  |  |  |
| 5 | Unguja | Kati | 5 |  |
| 6 | Arusha | Arusha CC | 4 |  |
| 7 | Arusha | Arusha DC | 3 |  |
| 8 | Dodoma | Dodoma CC | 2 |  |
|  |  |  |  |  |
| 9 | Mtwara | Mtwara MC | 11 |  |
| 10 | Mtwara | Masasi TC | 9 |  |
| 11 | Songwe | Tunduma TC | 10 |  |
|  |  |  |  |  |
| 12 | Mwanza | Ilemela MC | 11 |  |
| 13 | Mwanza | Nyamagana MC | 6 |  |
| 14 | Kigoma | Kigoma MC | 6 |  |
| 15 | Kigoma | Kasulu TC | 3 |  |
|  | Total markets visited |  | 108 |  |
